# ﻿Triploid female *Helophorusbrevipalpis* Bedel, 1881 in Provence, France, with additional data on C-banding in both triploid and diploid material, and discussion of chromosomal variation in *H.brevipalpis*

**DOI:** 10.3897/compcytogen.19.162685

**Published:** 2025-07-23

**Authors:** Robert B. Angus

**Affiliations:** 1 Department of Life Sciences (Insects), The Natural History Museum, London SW7 5BD, UK The Natural History Museum London United Kingdom

**Keywords:** C-banding, Coleoptera, France, *Helophorusbrevipalpis*, karyotypes, parthenogenesis, triploidy

## Abstract

Triploid female *Helophorusbrevipalpis* Bedel, 1881 are recorded from two localities in Provence, France. Their karyotypes are analysed using both chromosome morphology and C-banding. Their karyotypes appear to be identical with those of Spanish material recorded by Angus (1992) but show minor differences from Italian triploid material described by Angus, Jia (2020). Data on C-banding in English *H.brevipalpis* are given and chromosomal variation in *H.brevipalpis* is discussed.

## ﻿Introduction

*Helophorusbrevipalpis* is a very common beetle over much of Europe, noted for its swarming in mid- to late summer when it may land in great numbers on shiny surfaces. This behaviour was investigated in the Oxford (England) area by Fernando (1958). He found that in Spring and early Summer (March to early July) it colonised artificial habitats but was less common in glass traps. In later summer (July to October), however, it was far more abundant in the glass traps. He went on to show that this major dispersal phase was associated with newly emerged adults whose ovaries were in early stages of development. Landin (1958), working in central Sweden, investigated similar swarming in July. None of this work gave any hint that parthenogenesis might be involved.

The first indication that *H.brevipalpis* could be parthenogenetic came from the discovery of a females-only population in Logan Canyon, Utah, USA (Angus 1971). This, the only genuine North American record of the species, suggested that it had to be an introduction. Smetana (1985) caused some confusion by recording a male *H.brevipalpis* from Logan Meadows. However, he very kindly allowed me to examine this specimen, and it is in fact *H.orientalis* Motschulsky, 1860, which, though parthenogenetic over most of it range, has a bisexual population in the central Rocky Mountains (Angus et al. 2017; Angus and Jia 2020).

Parthenogenetic triploid *H.brevipalpis* were first identified from Spain, Provincia de León, where they were found along with bisexual diploids (Angus 1992). This record was followed by the discovery of parthenogenetic triploids in the Po valley in Italy (Angus 2009; Angus and Jia 2020). The French material reported in this paper represents a third area inhabited by triploids.

## ﻿Material and methods

**Figure 1.**
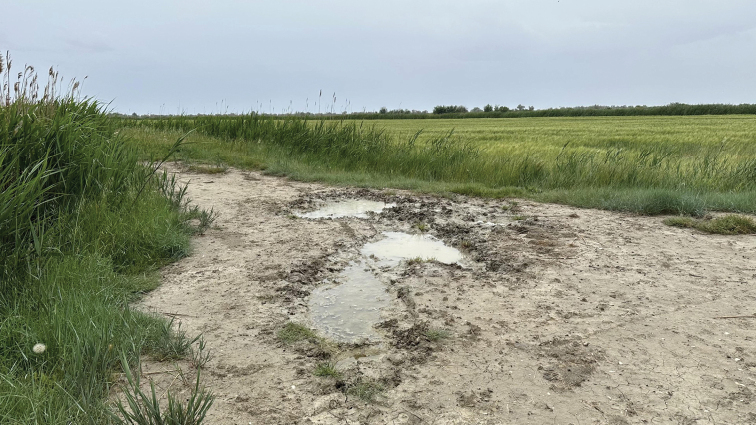
Collecting locality for triploid *H.brevipalpis* in the Camargue, France. Photo by Wenfei Liao.

The living material studied is as follows: 12 triploid females from a puddle near the Étang de Vaccarès in the Camargue, Bouches du Rhône, France, 43.537°N, 4.480°E leg. R.B. Angus & Wenfei Liao 11.v.2025 (Fig. 1); 1 triploid female from the Salagou River, Hérault, France, 43.631°N, 3.365°E leg. R.B. Angus 10.v.2025; 3 diploid females and 1 diploid male, puddle by Setley Pond, New Forest, Hampshire, England, 50.791°N, 1.572°W leg. R.B. Angus 30.v.2025. Older (dead) material reworked is one diploid male from the coastal road east of Rethymnon, Crete, Greece, 35.416°N, 24.548°E, leg. R.B. Angus 4–14.iv1996 and one diploid male from Israel, Golan, a pool at Bab el Hawa 33.143°N, 35.774°E, leg. Reuven Ortal, Jerusalem 23.i.1990. (not Abu Mashaq as given by Angus (1992)). Chromosome preparation from mid-gut and spermatogonial cells was included in the introductory discussion by Angus (2023), and a straightforward account is given by Angus (2006). Preparation from spermatogonial mitosis is complicated by the fact that adjacent cells in the spermatogonium synchronise their positions in the cell cycle and the membranes between adjacent cells may be weak or even absent. Fig. 3 shows mid-gut and spermatogonial cells of New Forest *H.brevipalpis* unbanded and with C-banding. C-banding was obtained by treatment for 7–8 minutes in a saturated solution of barium hydroxide (Ba(OH)_2_) at room temperature (about 20°C) followed by incubation in 2X SSC (salt-sodium citrate) at 55°C. Initial treatment for 10 min. in Ba(OH)_2_ followed by 1 hour in SSC at 60°C destroyed all stainability of the chromosomes. The first modification involved 5 minutes in Ba(OH)_2_ and 45 min. in SSC at 55°C, which not produce any banding, which was eventually achieved by treatment for a further 2 min. in Ba(OH)_2_ followed by a further 45 min. in SSC.

## ﻿Results

### ﻿French and Spanish triploids (Fig. 2a–d)

**Figure 2.**
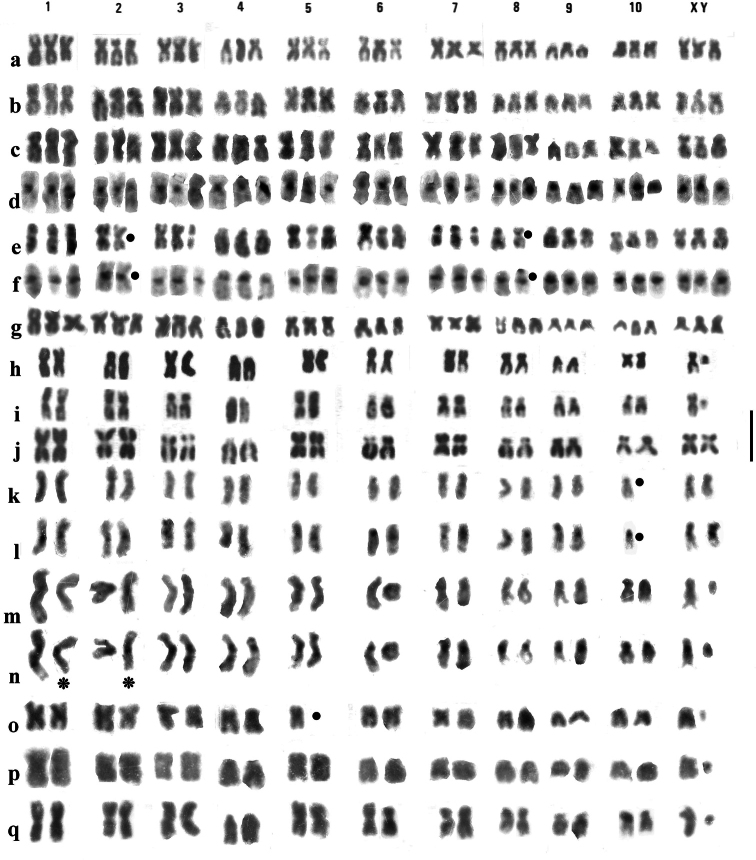
**a**–**q** mitotic chromosomes of *H.brevipalpis*, arranged as karyotypes. **a**–**g** triploid females **a** Spain, Provincia de León, Algadefe, Giemsa-stained **b** France, Bouches du Rhône, Camargue near Étang de Vaccarès, Giemsa-stained **c**, **d** France, Hérault, Marette River **c** Giemsa-stained **d** the same nucleus C-banded **e**, **f** Italy, Reggio Emilia, Sologno **e** Giemsa-stained **f** the same nucleus C-banded **g** Italy, Parma, Ponte Scipione, Giemsa-stained. **h**–**q** diploid nuclei **h**, **k**, **l, o–q** from mid-gut cells **i**, **j** from embryos **h** Spain, Provincia de León, El Cubo, Giemsa-stained **i**, **j** England, Somerset, Brean, Giemsa-stained **k**–**n** England, Hampshire, New Forest, puddle near Setley Pond **k, l** female **k** Giemsa-stained **l** the same nucleus C-banded **m**, **n** males, spermatogonia **m** Giemsa-stained **n** the same chromosomes C-banded **o, p** male, Israel, Golan, Bab el Hawa, Giemsa-stained **q** male, Greece, Crete, Rethymnon, Giemsa-stained. Missing chromosomes in **e**, **f**, **k**, **l**, **o** indicated by bold dots. Scale bar: 5μm.

The sequence of autosomes, arranged in order decreasing length, appears the same in French and Spanish material in terms of the position of chromosomes with different centromere positions. Autosomes 1, 3, 5 and 7 are clear metacentrics, 6, 8 and 10 are borderline metacentric/submetacentric, 2 is subacrocentric and 4 and 9 are acrocentric. The X chromosome is borderline submetacentric/subacrocentric and about the same length as autosomes 6 or 7. Angus (1992) placed the longest subacrocentric autosome as pair 3, but it is clearly longer than the one placed as pair 2 in the triploid, and more or less the same length in the diploid Spanish male (Angus 1992, figs 1, 4, 5), so it is placed as pair 2 in this paper. C-banding had not been shown in the original Spanish triploids but is here shown for the French material (Fig. 2d). The C-bands are confined to the centromere regions and are present in all the autosomes and the X chromosomes.

### ﻿Italian triploids (Fig. 2e–g)

The karyotypes of the Italian triploids (Angus and Jia 2020; Angus 2023) closely resemble those of the Spanish and French material, except there is no subacrocentric in position 3, which is clearly metacentric. The C-bands (Fig. 2f) are clear and strong (as in the French specimen shown in Fig. 2d).

### ﻿Diploids (Figs 2l, n, 3b, d)

Hitherto C-banding had not been demonstrated in diploid *H.brevipalpis*. The banding reported here is from Southern English material (Hampshire, New Forest). Moderate to weak centromeric C-bands are present on all the autosomes as well as on the X chromosome. The variation in the apparent strength of the C-bands is almost certainly associated with the difficulty experienced in obtaining these bands.

**Figure 3.**
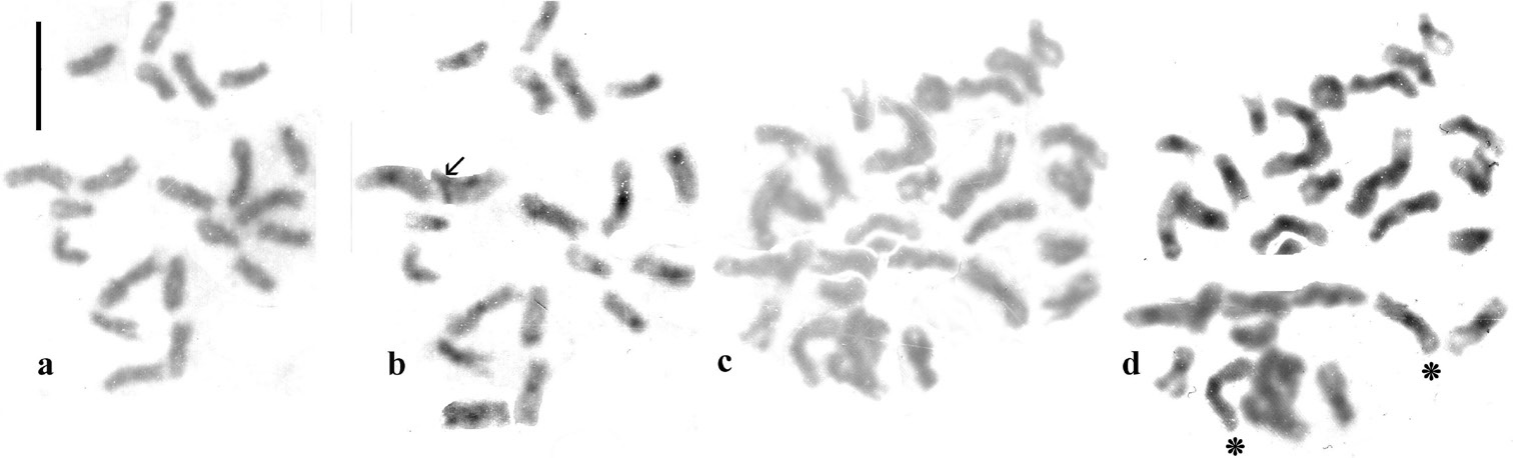
**a**–**d***H.brevipalpis*, England, Hampshire, New Forest puddle by Setley Pond, mitosis. **a**, **b** females, mid-gut cell **a** Giemsa-stained **b** the same nucleus C-banded. One C-banded chromosome (arrowed) has acquired some detritus in the course of C-banding treatment. This is shown, without the detritus, as chromosome 4, left replicate, in Fig. 2**l**. **c**, **d** males, mitotic chromosomes from spermatogonia **c** Giemsa-stained **d** the same chromosomes C-banded. In **c** the chromosomes are shown in their original positions in the cluster but in **b** they are shown separated along a possible boundary between two nuclei. All but two chromosomes are present in the upper part of the cluster, and the two from the lower part used to complete the karyotype are indicated by asterisks (*). Scale bar: 5μm.

## ﻿Discussion

Triploid *H.brevipalpis* appear to result in often abundant females-only populations. Thus, the superabundance of females in the Camargue population immediately aroused suspicion, but the triploidy of the single Hérault specimen came as a total surprise. The Spanish triploids, in León, are at the edge of the species’ range – *H.brevipalpis* appears to be absent from Galicia. The situation is Italy is less clear – certainly populations in the Po valley and adjacent territory are composed of triploid females, but males occur further south. The parthenogenetic population in Utah, USA (of unknown ploidy) may be the result of a female resorting to parthenogenesis in the absence of males, like female Komodo Dragons (*Varanuskomodoensis* Ouwens, 1912) in zoos. The diploid karyotypes shown here (Fig. 2h–q) show some slight variation. The Spanish specimen (Fig. 2h) has autosome 2 subacrocentric, as in the Spanish and French triploids (Fig. 2a–d), but in all the others this chromosome is metacentric, while pair 3, metacentric in the Spaniard, is clearly submetacentric in the others (Fig. 2i–q). Note that, as explained above, in this paper the subacrocentric chromosome in Spanish material, both diploid and triploid, is placed as pair 3. The Cretan specimen (*creticus* Kiesenwetter 1858, Fig. 2q) has autosome 6 clearly metacentric as against submetacentric/subacrocentric in the others. It is worth noting that the International Commission on Zoological Nomenclature (Opinion 1629) ruled that the name *brevipalpis* Bedel, 1881, though more recent than *creticus*, should have precedence if the names are regarded as synonymous. Angus (1988) sought to make sense of the morphological variation, both somatic and aedeagal, of *H.brevipalpis* and described a new subspecies, *H.brevipalpislevantinus* (Angus 1988) from disparate areas in Turkey, Lebanon and Iran. Discriminant functions analysis (Angus 1988, fig. 40a) grouped all these populations together and separate from the other populations involved. A dendrogram showing the relative distances between the centroids of the 18 groups (Angus 1988, fig. 40b) shows the three *levantinus* groups (16, 17 and 18) well clear of all the others except 13 (east of Lake Van), which was regarded as a possible intermediate between the two subspecies. This analysis points to its belonging to *levantinus*. The fairly clear separation of subspecies *levantinus* means that consideration of the material in this paper may be confined to subspecies *brevipalpis*. Examination of the dendrogram shows material from Northwest Europe, Italy and Greece to come out as a close group, along with that from Perm in the Urals, and with material from Turkey and Israel not far away. Material from Spain, Avignon and Odessa as well as that from northwest Iran and Alpine Georgia also comes together as a close group. This somewhat surprising juxtaposition of groups is a warning to expect surprises. Thus Angus (1984, 1985) shows markedly different aedeagal sizes for Rey’s types of *Helophorusmixtus* and *insignis*, both forms of *brevipalpis* described from Provence (Angus 1984, figs 52, 53), as well as *H.bulbipalpis* Kuwert (Angus 1984, fig. 54) from the Shetland Isles with an aedeagus distinctly larger than the lectotypes of *brevipalpis* and *creticus* (Angus 1984, figs 50, 51). Note that the illustrations are printed in part 1 of this work, but the relevant text is in part 2. The Russian illustrations of the aedeagi are better than the English ones. The work discussed here is now almost 40 years old and begs the question: what about DNA? Obviously, DNA sampling of multiple populations of *H.brevipalpis* would be highly desirable and may provide answers. However, some caution is needed here as the separation of various populations may have been too recent to show clear differences.
